# Healthcare Burden and Productivity Loss Due to Narcolepsy in Sweden

**DOI:** 10.3390/clockssleep7010008

**Published:** 2025-02-19

**Authors:** Anna Giertz, Johan Mesterton, Tanja Jakobsson, Stephen Crawford, Somraj Ghosh, Anne-Marie Landtblom

**Affiliations:** 1 Quantify Research, Hantverkargatan 8, 112 21 Stockholm, Sweden; annafornwall@hotmail.com; 2 Department of Learning, Informatics, Management and Ethics, Medical Management Centre, Karolinska Institutet, Tomtebodavägen 18 A, 171 77 Stockholm, Sweden; 3 Takeda Pharma AB, Lindhagensgatan 120, 112 51 Stockholm, Sweden; tanja.jakobsson@takeda.com; 4 Takeda Development Center Americas, Inc., Cambridge, MA 02142, USA; stephen.crawford@takeda.com (S.C.); som.ghosh@takeda.com (S.G.); 5 Department of Medical Sciences, Uppsala University, 751 05 Uppsala, Sweden; anne-marie.landtblom@uu.se; 6 Department of Biochemical and Clinical Sciences, Linköping University, 581 83 Linköping, Sweden

**Keywords:** cost, disability leave, healthcare resource utilization, narcolepsy, Pandemrix vaccination, pharmaceutical products, sick leave, Sweden, work productivity loss

## Abstract

Background: Narcolepsy impacts both patients and society, yet there is limited data on its socioeconomic consequences. Methods: This retrospective longitudinal cohort study used pseudonymized patient-level data from Swedish registers and included narcolepsy patients from January 2015–December 2019 and age–sex matched controls. All patients received an index date corresponding to their first narcolepsy diagnosis. Results: This study included 466 incident narcolepsy patients and 2330 matched controls. During the years studied, healthcare resource utilization was 2–5 times higher for incident narcolepsy patients compared to matched controls (*p* < 0.0001). Modafinil, stimulants, and antidepressants were prescribed more often to incident narcolepsy patients (*p* < 0.0001). Work productivity was significantly impacted, as incident narcolepsy patients took 7.0–10.5 more sick leave days than their matched controls (*p* < 0.0001) and had an average of 14.8 net days of disability leave (associated with indirect costs of EUR 1630) versus only 5.8 days among matched controls (EUR 638) during the year of the index (*p* = 0.027). After controlling for age, sex, and the Charlson comorbidity index, the odds of disability leave were 3.3 times higher in incident narcolepsy patients. Conclusions: This study provides evidence of the magnitude of the substantial societal economic burden due to narcolepsy in Sweden, evidenced by higher healthcare resource utilization and indirect costs.

## Simple Summary

This study examined patient data from Swedish healthcare registers between 2015 and 2019. It included 466 people who were newly diagnosed with narcolepsy and 2330 matched individuals without the condition. The researchers examined healthcare use, medications, and productivity loss related to narcolepsy. Individuals diagnosed with narcolepsy used healthcare services 2–5 times more than those without the condition. They were also more likely to be prescribed medications such as modafinil, stimulants, and antidepressants. They took more sick leave, averaging 7–10 more days than the matched controls. They also had higher disability leave, with an average of 14.8 days (costing EUR 1630), compared to 5.8 days (costing EUR 638) for the controls. The chances of taking disability leave were 3.3 times higher in people diagnosed with narcolepsy than in those without the condition. This study shows that narcolepsy in Sweden leads to higher healthcare resource use and significant costs due to lost productivity. This highlights the substantial economic burden of the condition on both individuals and society.

## 1. Introduction

Narcolepsy is a chronic neurological disorder characterized by excessive daytime sleepiness, sleep paralysis, hallucinations at sleep onset (hypnagogic) and/or offset (hypnopompic), disturbed night-time sleep, and is mostly associated with cataplexy [1]. In the latest text revision of the *International Classification of Sleep Disorders*, 3rd Edition, narcolepsy has been classified as narcolepsy type 1 (NT1) or narcolepsy type 2 (NT2) [2,3]. NT1 is characterized by the presence of cataplexy and a profound loss of orexin-producing neurons in the lateral hypothalamus. In contrast, individuals with NT2 do not exhibit cataplexy or suffer from orexin deficiency [2,3,4]. Patients with narcolepsy often have comorbid conditions, including sleep disorders (such as insomnia, obstructive sleep apnea, and restless leg syndrome), mood disorders (like depression, anxiety, and suicidal thoughts), and pain conditions (such as myalgia, carpal tunnel syndrome, fibromyalgia, migraines, and chronic pain syndrome) [5,6,7]. Notably, individuals with NT1 frequently experience symptoms of eating disorders, particularly night eating syndrome [8].

Reports on prevalence from different populations globally range from 0.2 to 65.4 per 100,000 individuals, except for one study that reported a prevalence of 79.4 per 100,000 individuals, which is an outlier owing to the case ascertainment algorithm used, which resulted in a greater sensitivity and lower specificity compared to other reported studies [9,10]. The wide variation in prevalence may be due to differences in geographies, age, study design, study periods (ranging from 1960 to 2018), and diagnostic criteria [10,11,12,13]. In the West, the estimated prevalence ranges from 20 to 60 per 100,000 individuals, which would correspond to an estimated range of 3500 to 4500 patients in Sweden [14]. In Germany, which, in many aspects, is similar to Sweden, Kallweit et al. recently reported a prevalence of 17.9 per 100,000 individuals [15]. However, a recent study by Gauffin et al. identified only 2508 prevalent individuals with narcolepsy in specialist care using the Swedish National Patient Register [12]. The development of narcolepsy following the administration of the Pandemrix vaccine also led to an increase in incidence in various European countries (Finland, France, Ireland, the Netherlands, Norway, Sweden, and the United Kingdom), where it was widely used between 2009 and 2010. Studies have estimated a 5- to 14-fold increased risk of developing childhood (and adolescent) narcolepsy and a 2- to 7-fold increase in adults during the first year after vaccination [16].

Narcolepsy is associated with a substantial medical and socioeconomic burden and high healthcare costs [1,17,18], with direct medical costs approximately twice as high as the general population of the United States (USD 11,702 versus USD 5261) [17], along with significantly higher costs related to work absenteeism (USD 12,839 versus USD 7631) and presenteeism (USD 7013 versus USD 4987) [18]. Moreover, patients with narcolepsy incur approximately two times higher inpatient admissions (0.15 versus 0.08), emergency department visits without admission (0.3 versus 0.2), hospital outpatient visits (2.80 versus 1.40), other outpatient services (7.0 versus 3.2), and physician visits (11.1 versus 5.60) compared to matched controls [17]. They also had 3-fold higher annual short-term disability costs (USD 876 versus USD 292) than matched controls [17] with more than 1.7-fold greater long-term disability [18].

A recent Swedish study assessing healthcare resource utilization (HCRU) for patients with narcolepsy compared with the general population found that narcolepsy patients had higher annual average hospitalizations (0.2 versus 0.1) and outpatient visits (2.6 versus 1.1) than the general population; these data were taken from a crude post hoc analysis [12]. Narcolepsy inpatient care can be motivated by investigations like polysomnography or cardiac investigations [15] or by trauma caused by cataplexy or traffic accidents [19].

There are only a few studies that have assessed the health-related burden and socioeconomic consequences of narcolepsy in Sweden [12,20]. The present study aimed to describe the characteristics of patients with narcolepsy, their HCRU, and work-related outcomes by conducting a retrospective non-interventional study in Sweden based on secondary data for the period between 2010 and 2020.

## 2. Results

This study included 466 incident narcolepsy patients and 2330 matched controls. During the years studied, healthcare resource utilization was 2–5 times higher for incident narcolepsy patients compared to matched controls (*p <* 0.0001). Modafinil, stimulants, and antidepressants were prescribed for incident narcolepsy patients significantly more often than for matched controls (*p <* 0.0001). Work productivity was also significantly impacted: incident narcolepsy patients took 7.0–10.5 more sick leave days than matched controls (*p <* 0.0001) and had an average of 14.8 net days of disability leave (associated with indirect costs of EUR 1630) versus only 5.8 days among matched controls (EUR 638) during the year of the index (*p* = 0.027). After controlling for age, sex, and the Charlson comorbidity index, the odds of taking disability leave for incident narcolepsy patients were 3.3 times higher compared to matched controls.

### 2.1. Demographic Profile and Characteristics of Incident Narcolepsy Patients and Matched Controls

A total of 466 incident narcolepsy patients and 2330 matched controls were included in this study. Incident narcolepsy patients and controls were matched on age (mean age of incident narcolepsy patients and matched controls at index: 31.0 years) and sex (incident narcolepsy patients: 59.2% females; matched controls: 60.4% females) and were therefore very similar in these demographics. Almost two-thirds (62.9%) of incident narcolepsy patients were under the age of 30, including 28.3% who were 18 years of age or younger (versus 27.7% in matched controls) and 34.6% between the ages of 19 and 29 years (versus 33.7% in matched controls). The cohorts were similar in terms of employment status, with around half being employed at the time of the index (51.3% among incident narcolepsy patients and 50.8% among matched controls) (Table 1). Both cohorts were also comparable in terms of education level (university level: 39.1% versus 39.3% among incident narcolepsy patients and matched controls, respectively).

### 2.2. Healthcare Resource Utilization

#### Healthcare Contacts

**Figure 1:**
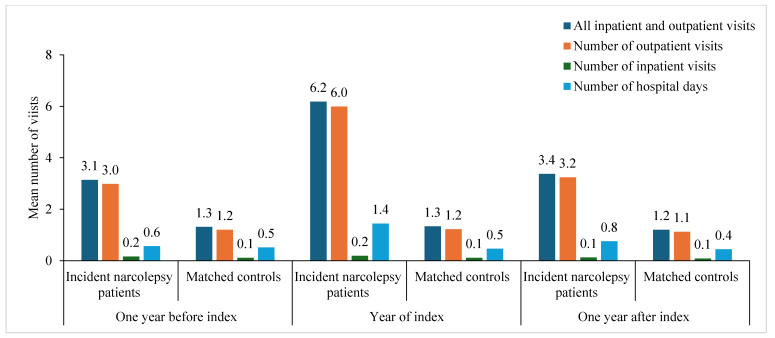
Healthcare contacts (outpatient and inpatient care) one year before the index, during the year of the index, and one year after the index among incident narcolepsy patients identified in specialist care data and matched controls in Sweden between the years 2015 and 2020.

The mean number of all inpatient and outpatient visits was statistically significantly higher (*p <* 0.0001) among incident narcolepsy patients compared to matched controls in the year before the index (3.1 versus 1.3), year during the index (6.2 versus 1.3), and year after the index (3.4 versus 1.2) (Figure 1). The difference in outpatient visits was statistically significant for all years (*p <* 0.0001) and for inpatient visits during the year of the index (*p* = 0.007). During the year of the index, incident narcolepsy patients had, on average, 6.2 visits, of which 0.2 were hospitalizations, compared to 1.3 and 0.1 among matched controls (Supplementary Table S1).

All comparisons between narcolepsy incident patients and matched controls are statistically significantly different (*p* < 0.05) except 1. number of inpatient visits one year before the index and after the index and 2. number of hospital days one year before the index, one year during the index, and one year after the index.

### 2.3. Number of Medications

**Figure 2:**
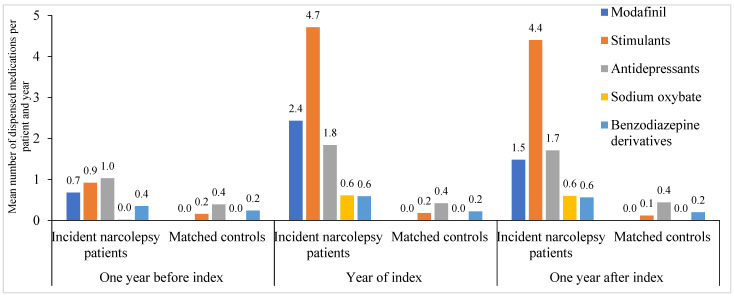
Medications used one year before the index, during the year of the index, and one year after the index by incident narcolepsy patients identified in specialist care data and matched controls in Sweden between the years 2015 and 2020.

Modafinil, stimulants, and antidepressants were statistically significantly more used among incident narcolepsy patients compared with matched controls in all years (*p <* 0.0001) (Figure 2). During the year before the index, the most used medications among incident narcolepsy patients were antidepressants (20.8%), followed by modafinil (16.1%) and stimulants (14.8%). However, during the index year and the year following, stimulants were used most frequently (54.5% and 57.0%, respectively). From the year before the index to the year of the index, the use of modafinil increased from 16.1% to 53.4%, and antidepressants from 20.8% to 39.7% (Supplementary Table S1).

### 2.4. Work Productivity and Indirect Costs

#### 2.4.1. Sick Leave

During the year before the index, 25.3% of the incident narcolepsy patients took sick leave compared to 14.0% of the matched controls. The proportion of incident narcolepsy patients taking sick leave increased to 40.1% in the year of the index and remained between 30 and 40% throughout the study period. However, the percentage of matched controls taking sick leave decreased to 10.3% in the year after the index.

During the year of index, incident narcolepsy patients had, on average, 15.0 net sick leave days, compared to 4.6 net sick leave days among matched controls (*p <* 0.0001). This was associated with indirect costs of EUR 1657 compared to EUR 510 for the incident narcolepsy patients and matched controls, respectively (Table 2).

#### 2.4.2. Disability Leave

During the year before the index, 6.4% of incident narcolepsy patients had disability leave, and this share increased in the year of the index to 8.6% and remained at a high level in the year after the index (8.8%). However, only 3.5–4.0% of matched controls took disability leave throughout the study period. The mean days of net disability leave were consistently higher for incident narcolepsy patients compared to matched controls throughout the years of the study, and the difference was statistically significant for the year after index (*p* = 0.027) and the year after index (*p* = 0.035). During the year of index, incident narcolepsy patients had, on average, 14.8 net days of disability leave, compared to 5.8 days among matched controls. This was associated with indirect costs of EUR 1630 and EUR 638 for incident narcolepsy patients and matched controls, respectively (Table 2).

#### 2.4.3. Odds of Disability Leave

Compared with patients without narcolepsy, patients with narcolepsy showed 3.3 times higher odds of taking disability leave (odds ratio: 3.3; 95% confidence interval [CI]: 3.2–3.4) after controlling for age, sex, and the Charlson comorbidity index (Table 3).

## 3. Discussion

This study captured patients with a diagnosis of narcolepsy after the H1N1 pandemic when the incidence of narcolepsy increased in Sweden in response to the Pandemrix vaccination [21,22,23]. Wijnans et al., 2013, reported that children and adolescents aged 5–19 years showed an increase in incidence rate after the start of vaccination compared with the period before the start of vaccination campaigns in Sweden [24].

This study describes the characteristics of patients diagnosed with narcolepsy between 2015 and 2019, along with their HCRU and work-related outcomes, and compares them to matched controls. The incident narcolepsy patients and matched controls were well matched in age and sex. One-third (34.6%) of incident narcolepsy patients were between 19 and 29 years of age. This could be due to the delayed diagnosis of narcolepsy in children and adolescent patients, some of whom may have developed narcolepsy after receiving the Pandemrix vaccine. Overall, the use of healthcare contacts and medications was higher in incident narcolepsy patients than in matched controls, which is in line with previous studies [17,18]. This suggests that narcolepsy poses a significant burden on the healthcare system as well as society. Such a high HCRU rate among patients with narcolepsy is also associated with higher treatment costs.

In Sweden, many of the medications used to treat narcolepsy do not have an indication for this use. These treatments are covered by the benefit scheme for other indications but are prescribed off-label for narcolepsy treatment. However, studies report that a meaningful share of patients with narcolepsy remain untreated, which might be due to patients receiving medications that are not included in this study. According to Gauffin et al., 2022, a few of the drugs used in Sweden (atomoxetine, dexamphetamine, and lisdexamphetamine) are not being used for narcolepsy in some other countries, suggesting national/geographical differences in drug utilization [12]. According to European guidelines and expert statements on the management of narcolepsy (2021), the first-line therapy for excessive daytime sleepiness as the main symptom is monotherapy with modafinil, pitolisant, or solriamfetol. Second-line treatment involves transitioning to a combination therapy, which could include pitolisant and modafinil or solriamfetol, or switching to a combination of sodium oxybate and any wake-promoting agent. Alternatively, patients may change to monotherapy with sodium oxybate, methylphenidate, or amphetamines [25]. Modafinil, pitolisant, and sodium oxybate have also been approved by the European Medicines Agency [26,27,28,29]. Modafinil is one of the most used drugs in the present study, and this is in line with the European guidelines [25] and the previous study by Gauffin et al., 2022 [12]. Solriamfetol was not available in Sweden during the study period, and the Swedish reimbursement system does not cover pitolisant and sodium oxybate since their cost-effectiveness has not been determined. A possible explanation for the lower-than-expected usage of narcolepsy-related medications might be the hesitance towards sodium oxybate being an addictive drug [30] or the limited availability of updated medical reports on pitolisant reaching general neurologists as well as the incapacity of patients to pay for narcolepsy-related medications if compensation is not granted.

While the health-related burden of narcolepsy has been well-documented, few studies have also focused on its societal costs, including work productivity loss and associated indirect costs. These studies reported that the indirect costs related to work productivity loss are substantially higher among patients with narcolepsy [17,18,31]. In line with previous literature, our study showed a consistent pattern of a greater socioeconomic burden in incident narcolepsy patients than in matched controls. To our knowledge, none of the prior studies have reported the societal costs that arise from the work productivity loss associated with narcolepsy in Sweden.

A higher proportion of patients had sick and disability leave compared with the matched controls. Previous studies suggest that there are often major cognitive problems related to narcolepsy that limit the daily function [32]. Since sick leave periods are only captured after 14 days, these data are likely an underestimate.

These findings highlight the significant burden narcolepsy is associated with, both in terms of HCRU and productivity losses, with increased sick leave and disability days contributing to indirect costs. This highlights the need for greater investment in early diagnosis, ongoing management, and access to effective treatments. Allocating more resources to narcolepsy care and implementing personalized treatment plans, including optimized pharmacological therapies, could improve patient outcomes and reduce healthcare utilization. By managing symptoms more effectively, patients may experience fewer sick days and disability leave, minimizing productivity losses.

## 4. Methods

### 4.1. Study Design and Patient Population

This was a retrospective longitudinal cohort study which used pseudonymized patient-level data from Swedish registers, namely the National Patient Register (includes data for specialist inpatient care [available from 1964 onwards] and specialist outpatient care [available from 1997 onwards]) [33], the Prescribed Drug Register (includes date of prescription and dispensation, formulation, amount dispensed, defined daily dose, and others from July 2005 onwards) [34], the Longitudinal integrated database for health insurance and labor market studies (from 1990 onwards) [35], and the Swedish Social Insurance Register/Microdata for social insurance analysis [36].

The Swedish registers cover the entire Swedish population of approximately 10 million individuals. Patients are included in these registers from the time of birth until the time of death or emigration. This prevents loss to follow-up and ensures that the cohorts are representative of the general population, offering high quality and completeness of data for decision-making.

**Figure 3:**
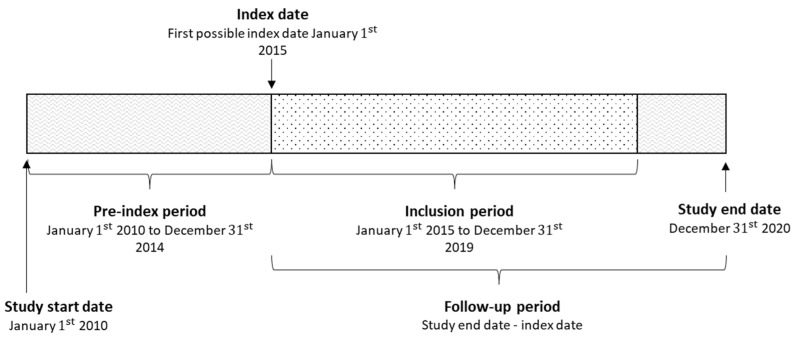
Schematic overview of the study period.

The study period ranged from 1 January 2010 to 31 December 2020 (Figure 3). Patients of all ages with a recorded primary or secondary diagnosis of narcolepsy (ICD-10 codes: G47.4, G47.4A, G47.4B, G47.4W, and G47.4X) in specialist inpatient or outpatient care between 1 January 2015 and 31 December 2019 were identified. In order to capture incident cases, patients with at least two diagnoses of narcolepsy, of which at least one was primary, in specialist care between the same time period but with no diagnosis of narcolepsy in the five years prior to the identification period (hereafter referred to as ‘incident narcolepsy patients’) were included in this study.

Incident narcolepsy patients were matched on age and sex at a 1:5 ratio to the general population. The matched controls consisted of individuals without a diagnosis of narcolepsy during the entire study period.

### 4.2. Study Period, Follow-Up, and Look-Back Periods

All patients received an index date corresponding to the date of their first narcolepsy diagnosis in the inclusion period. The matched controls received the same index date as their matched case. The look-back period consisted of three years prior to the index date, which was used to identify comorbidities. Patients were censored from the study in the event of death or end of the study period. All patients had follow-ups from the index date until death or the end of the study period.

### 4.3. Study Variables and Definitions

Demographic profile and characteristics of the incident narcolepsy patients and matched controls: The age, sex, employment status, and education level of incident narcolepsy patients and matched controls were investigated. Employment status (whether employed or not) was based on the register definition and available for patients between 16 and 74 years of age. Educational level was defined as the highest completed education at the index.

*HCRU:* The number of healthcare contacts (including specialized outpatient visits and hospitalizations) and dispensed medications were assessed one year prior to the index, during the year of the index, and one year after the index. Inpatient visit was defined as a hospital visit with admission (with or without an overnight stay). A specialized outpatient visit was defined as any outpatient visit to a specialist.

*Work absence and indirect costs:* Absence from work and associated costs related to sick leave and disability leave were assessed in incident narcolepsy patients and were compared with matched controls. Sick leave, as well as disability leave was defined based on days reported to the Swedish Social Insurance Agency. In Sweden, sick leave is only granted to employed individuals, and reporting to the agency is only mandatory after 14 days of leave, i.e., data on sick leave does not capture sick leave periods ≤ 14 days. As a result, analyses based on data on sick leave underestimate the actual sick leave.

Disability leave is granted to anyone who is considered unable to work/study, regardless of their employment status, and data covers all disability leave days (from Day 1).

Disability leave may be granted for a shorter period or the remainder of life. Indirect cost was defined as the net days not working × daily wage. The daily wage was calculated using the average wage per calendar year × 12/365.25. The daily wage was consumer price index-adjusted, and costs were presented in EUR as of the year 2020.

The odds of disability leave were calculated by taking the ratio of the odds of disability leave occurring in patients with narcolepsy and the odds of disability leave occurring in patients without narcolepsy. The outcome of the logistic regression was disability leave at any time during the study period. The analysis included the period one to three years from the index, which is the same period that was used for the descriptive analyses, and the full study population (patients and controls).

### 4.4. Data Analysis

Data management and statistical analyses were performed using R version 4.0 and Stata version 16. A two-sample *t*-test at a 95% confidence level was used to compare means between the cohorts. Odds ratios were estimated using conditional logistic regression.

## 5. Conclusions

The present study provides nationwide data on the health-related contacts and socioeconomic consequences of narcolepsy in Sweden. Incident narcolepsy patients, compared with matched controls, experienced more medical contacts with greater use of pharmaceuticals and took a higher number of sick and disability leaves. Higher resource use and work productivity loss, in turn, contributed to an increased burden of indirect costs among incident narcolepsy patients compared with matched controls.

## 6. Limitations

This was an observational retrospective study, so inference on causal mechanisms is not possible. The findings of the present study were based on Swedish data and are therefore not directly generalizable to other countries, as treatment practices, costs, compensation/reimbursement schemes, and underlying characteristics in other populations may differ. Additionally, this study only included specialist inpatient and outpatient visits, excluding general practitioner or primary care visits, which may result in an underestimation of the true burden of narcolepsy. Moreover, data on sick leave did not include the first 14 days of sick leave; hence, there is an underestimation of work productivity losses. Finally, the inability to distinguish between NT1 and NT2 limits the generalizability of the findings, as these subtypes differ in their epidemiology and treatment approaches.

## Figures and Tables

**Table 1:** Demographic profile of incident narcolepsy patients identified in specialist care data and matched controls in Sweden between the years 2015 and 2020.

	Percentage of Patients (%)	
Patient Demographics at Index ^1^	Incident Narcolepsy Patients (*n* = 466)	Matched Controls (*n* = 2330)
Male	40.8	39.6
Female	59.2	60.4
Age at index, years (mean [SD])	31.0 (18.4)	31.0 (18.5)
Age distribution at index		
≤18 years	28.3	27.7
19–29 years	34.6	33.7
30–39 years	10.7	11.0
40–49 years	11.2	11.3
50–59 years	3.9	3.5
60–69 years	5.2	5.7
70–79 years	4.3	4.7
≥80 years	1.9	2.4
Employment status ^2^		
Unemployed (16–74 years)	48.7	49.2
Employed (16–74 years)	51.3	50.8
Unemployed ^3^ (18–<65 years)	40.4	40.6
Employed ^3^ (18–<65 years)	59.6	59.4
Education level ^4^		
Pre-high school (Förgymnasial)	19.5	23.6
High school (Gymnasial)	8.8	10.8
University	39.1	39.3
PhD	17.4	16.6

^1^ The index date is a proxy for diagnosis (for incident narcolepsy patients, the index date is the first diagnosis of narcolepsy within the study period with a prior diagnosis-free period of 5 years); ^2^ in November of the index year; ^3^ total number of incident narcolepsy patients and matched controls between 18 and <65 years of age are 314 and 1528, respectively; ^4^ highest education level achieved at the time of the index. PhD, Doctor of Philosophy; SD, standard deviation.

**Table 2:** Sick leave and disability leave taken one year before the index, during the year of the index, and one year after the index by incident narcolepsy patients identified in specialist care data and matched controls in Sweden between the years 2015 and 2020.

	One Year Before Index					Year of Index					One Year After Index				
Sick Leave and Disability Leave	Incident Narcolepsy Patients (*n* = 466)		Matched Controls (*n* = 2330)			Incident Narcolepsy Patients (*n* = 466)		Matched Controls (*n* = 2330)			Incident Narcolepsy Patients (*n* = 466)		Matched Controls (*n* = 2330)		
Sick leave															
	N ^1^ (%)	Mean ^2^	N ^1^ (%)	Mean (control)	*p*-value	N ^1^ (%)	Mean ^2^	N ^1^ (%)	Mean (control)	*p*-value	N ^1^ (%)	Mean ^2^	N ^1^ (%)	Mean (control)	*p*-value
Number of gross sick leave days	118 (25.3)	14.6	326 (14.0)	5.2	<0.0001	187 (40.1)	22.9	310 (13.3)	5.8	<0.0001	173 (37.1)	20.1	240 (10.3)	4.42	<0.0001
Number of net sick leave days	118 (25.3)	11.5	326 (14.0)	4.2	0.001	187 (40.1)	15.0	310 (13.3)	4.6	<0.0001	173 (37.1)	12.6	240 (10.3)	3.59	<0.0001
Total indirect costs (EUR)	118 (25.3)	1258	326 (14.0)	452	0.001	187 (40.1)	1657	310 (13.3)	510	<0.0001	173 (37.1)	1401	240 (10.3)	398	<0.0001
Disability leave															
Number of gross disability leave days	30 (6.4)	13.4	93 (4.0)	7.5	0.122	40 (8.6)	16.8	93 (4.0)	6.7	0.015	41 (8.8)	17.0	82 (3.5)	6.9	0.020
Number of net disability leave days	30 (6.4)	12.4	93 (4.0)	6.8	0.148	40 (8.6)	14.8	93 (4.0)	5.8	0.027	41 (8.8)	15.2	82 (3.5)	6.0	0.035
Total indirect costs (EUR)	30 (6.4)	1355	93 (4.0)	746	0.149	40 (8.6)	1630	93 (4.0)	638	0.027	41 (8.8)	1683	82 (3.5)	668	0.035

^1^ Number of patients with at least one sick leave period; ^2^ number of days/cost per patient and year.

**Table 3:** Odds of disability leave.

Outcome: Disability Leave	Full Study Population		
	Odds Ratio	95% CI	*p*-Value
Covariates			
Intercept	0.1	0.1–0.2	<0.0001
Age (continuous)	1.0	1.0–1.0	<0.0001
Male (ref: female)	0.7	0.7–0.8	<0.0001
Narcolepsy diagnosis (ref: no narcolepsy diagnosis)	3.3	3.2–3.4	<0.0001
Charlson comorbidity index (integer)	1.0	1.0–1.0	<0.0001

CI, confidence interval.

## Data Availability

No patient-level data can be made available to other researchers due to Swedish data legislation.

## Supplementary Materials

The following supporting information can be downloaded at https://www.mdpi.com/article/10.3390/clockssleep7010008/s1. Table S1: Healthcare contacts and medications used one year before index, during the year of index and one year after index by incident narcolepsy patients identified in specialist care data and matched controls in Sweden between year 2015–2020.
