# Correction: Giertz et al. Healthcare Burden and Productivity Loss Due to Narcolepsy in Sweden. *Clocks & Sleep* 2025, *7*, 8

**DOI:** 10.3390/clockssleep7020027

**Published:** 2025-05-28

**Authors:** Anna Giertz, Johan Mesterton, Tanja Jakobsson, Stephen Crawford, Somraj Ghosh, Anne-Marie Landtblom

**Affiliations:** 1 Quantify Research, Hantverkargatan 8, 112 21 Stockholm, Sweden; annafornwall@hotmail.com; 2 Department of Learning, Informatics, Management and Ethics, Medical Management Centre, Karolinska Institutet, Tomtebodavägen 18 A, 171 77 Stockholm, Sweden; 3 Takeda Pharma AB, Lindhagensgatan 120, 112 51 Stockholm, Sweden; tanja.jakobsson@takeda.com; 4 Takeda Development Center Americas, Inc., Cambridge, MA 02142, USA; stephen.crawford@takeda.com (S.C.); som.ghosh@takeda.com (S.G.); 5 Department of Medical Sciences, Uppsala University, 751 05 Uppsala, Sweden; anne-marie.landtblom@uu.se; 6 Department of Biochemical and Clinical Sciences, Linköping University, 581 83 Linköping, Sweden

In the original publication [1], there was a mistake in Figure 1 as published. Currently, the *X*-axis shows only “Year before index” for all patients. However, it should include three categories: “One Year before index”, “Year of index”, and “One year after index”. The corrected Figure 1 appears below. The authors state that the scientific conclusions are unaffected. This correction was approved by the Academic Editor. The original publication has also been updated.

## Figures and Tables

**Figure 1:**
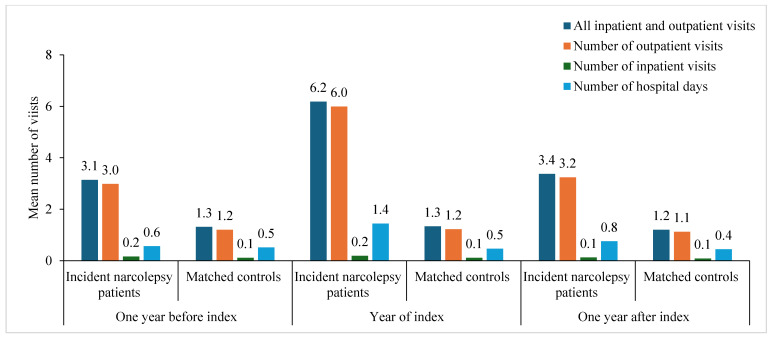
Healthcare contacts (outpatient and inpatient care) one year before the index, during the year of the index, and one year after the index among incident narcolepsy patients identified in specialist care data and matched controls in Sweden between the years 2015 and 2020.
