# Supplementary material for: Healthcare Burden and Productivity Loss Due to Narcolepsy in Sweden
Source: Clocks Sleep. 2025 Feb 19;7(1):8. doi: 10.3390/clockssleep7010008 (PMC11843934; doi:10.3390/clockssleep7010008)
Supplement: Supplementary file 1 [file clockssleep-07-00008-s001.zip › clockssleep-3416728-supplementary.pdf]

Supplementary file

Supplementary Table S1. Healthcare contacts and medications used one year before index, during the year of index and one year after index by incident narcolepsy patients identified in specialist care data and matched controls in Sweden between year 2015–2020

	**One year before index**					**Year of index**					**One year after index**				
**Healthcare contacts and medications**	**Incident narcolepsy patients (n=466)**			**Matched controls (n=2,330)**		**Incident narcolepsy patients (n=466)**			**Matched controls (n=2,330)**		**Incident narcolepsy patients (n=463)**			**Matched controls (n=2,318)**	
**Inpatient and outpatient care**															
	N[1]	%	Mean[2]	Mean (control)	*p*-value	N[1]	%	Mean[2]	Mean (control)	*p*-value	N[1]	%	Mean[2]	Mean (control)	*p*-value
All inpatient and outpatient visits	367	78.8	3.1	1.3	<0.0001	466	100.0	6.2	1.3	<0.0001	368	79.5	3.4	1.2	<0.0001
**Outpatient care**															

Number of outpatient visits	367	78.8	3.0	1.2	<0.0001	466	100.0	6.0	1.2	<0.0001	367	79.3	3.2	1.1	<0.0001
**Inpatient care**															
Number of inpatient visits	49	10.5	0.2	0.1	0.096	59	12.7	0.2	0.1	0.007	42	9.1	0.1	0.1	0.159
Number of hospital days	46	9.9	0.6	0.5	0.795	53	11.4	1.4	0.5	0.220	36	7.8	0.8	0.4	0.406
**Medications**															
	N[3]	%	Mean[4]	Mean (control)	*p*-value	N[3]	%	Mean[4]	Mean (control)	*p*-value	N[3]	%	Mean[4]	Mean (control)	*p*-value
Modafinil	75	16.1	0.7	0.0	<0.0001	249	53.4	2.4	0.0	<0.0001	149	32.2	1.5	0.0	<0.0001
Stimulants	69	14.8	0.9	0.2	<0.0001	254	54.5	4.7	0.2	<0.0001	264	57.0	4.4	0.1	<0.0001

Pitolisant	≤5	-	0.0	0.0	0.318	≤5	-	0.0	0.0	0.195	≤5	-	0.0	0.0	0.059
Antidepressants	97	20.8	1.0	0.4	<0.0001	185	39.7	1.8	0.4	<0.0001	162	35.0	1.7	0.4	<0.0001
Sodium oxybate	≤5	-	0.0	0.0	0.128	45	9.7	0.6	0.0	<0.0001	47	10.2	0.6	0.0	<0.0001
Benzodiazepine derivatives	29	6.2	0.4	0.2	0.385	40	8.6	0.6	0.2	0.065	32	6.9	0.6	0.2	0.136
Melatonin receptor agonists	17	3.7	0.1	0.1	0.382	51	10.9	0.4	0.1	<0.0001	47	10.2	0.3	0.1	<0.0001
Other hypnotics and sedatives	12	2.6	0.1	0.1	0.963	13	2.8	0.1	0.1	0.276	19	4.1	0.1	0.1	0.167
Hypnotics	≤5	-	0.0	0.1	0.026	10	2.2	0.1	0.1	0.224	9	1.9	0.1	0.1	0.406
Baclofen	≤5	-	0.0	0.0	0.332	≤5	-	0.0	0.0	0.312	≤5	-	0.1	0.0	0.140

[1]Number of patients with at least one visit; [2]Number of visits; [3]Number of patients with at least one dispensation of medication;

[4]Number of dispensed medications per patient and year
